# The Effect of Spatial Working Memory Deterioration on Strategic Visuomotor Learning across Aging

**DOI:** 10.1155/2015/512617

**Published:** 2015-07-28

**Authors:** Luis A. Uresti-Cabrera, Rosalinda Diaz, Israel Vaca-Palomares, Juan Fernandez-Ruiz

**Affiliations:** ^1^Program in Neuroethology, University of Veracruz, 91190 Xalapa, VER, Mexico; ^2^Laboratory of Neuropsychology, Department of Physiology, Faculty of Medicine, National Autonomous University of Mexico, Coyoacán, 04510 Mexico City, DF, Mexico

## Abstract

*Objective*. To evaluate the effect of age-related cognitive changes in a visuomotor learning task that depends on strategic control and contrast it with the effect in a task principally depending on visuomotor recalibration. *Methods*. Participants performed a ball throwing task while donning either a reversing dove prism or a displacement wedge prism, which mainly depend on strategic control or visuomotor recalibration, respectively. Visuomotor performance was then analysed in relation to rule acquisition and reversal, recognition memory, visual memory, spatial planning, and spatial working memory with tasks from the Cambridge Neuropsychological Test Automated Battery (CANTAB). *Results*. The results confirmed previous works showing a detrimental effect of age on visuomotor learning. The analyses of the cognitive changes observed across age showed that both strategic control and visuomotor recalibration had significant negative correlations only with the number of errors in the spatial working memory task. However, when the effect of aging was controlled, the only significant correlation remaining was between the reversal adaptation magnitude and spatial working memory. *Discussion*. These results suggest that spatial working memory decline across aging could contribute to age-dependent deterioration in both visuomotor learning processes. However, spatial working memory integrity seems to affect strategic learning decline even after controlling for aging.

## 1. Introduction

Visuomotor adaptation, a kind of motor learning, involves the capacity to adjust motor performance to reduce systematic errors during visually guided motor tasks. This is carried out by the modification of previously learned visuomotor transformations and its sensory outcomes [1–3]. At least two different processes have been proposed to underlie visuomotor adaptation [4–6]. One process is an implicit recalibration of the sensorimotor system, which refers to the transformation of visuomotor coordinates into proprioceptive-motor coordinates. This is believed to be a largely automatic process that fits within the definition of procedural learning [7]. A second process involves strategic resources, which include cognitive schemes and anticipation and movement corrections based on visual feedback and feed forward strategies to compensate for a perturbation [6, 8]. Though different, these two processes can occur simultaneously [9–13].

While the implicit recalibration process seems to be driven by a sensory-prediction error mechanism [14], it is unclear what mechanisms are involved during the strategic control process. The modification of the visuomotor transformation can engage cognitive resources for error encoding and visuomotor impairment updating [15–17]. It has also been suggested that the use of cognitive strategies relies on spatial working memory [8, 13, 17–19] and cognitive flexibility [20]. However, the cognitive participation during the adaptive process to external perturbations such as visuomotor rotations seems to be present mainly during an initial stage, that is, the early trials, while the later stage is largely automatic [17, 21].

Previous studies have reported that the role of cognition in visuomotor learning may be differentially affected by aging [16, 22], which could be related to the well-known decline in several cognitive domains in older adults (i.e., [3, 23–27]). For example, it has been found that failure to effectively engage spatial working memory processes contributes to age-related deficits in rotational visuomotor learning [22]. Also, it has been reported that response speed and decision-making are related to degrade adaptive improvement in seniors [16].

A paradigm that has been useful to study the effect of aging on visuomotor learning is prism adaptation. In these tasks, the motor system responds to new coordinates imposed by prisms that perturb the visual field [7, 28]. Different types of prism perturbations have been studied, including wedge prisms that result in a lateral displacement of the visual field [6, 7, 29, 30] and dove prisms that result in a one axis reversal of the visual field [9, 11]. The critical difference between these perturbations is the magnitude of the contribution of the procedural and the strategic processes to solve each task. While the error reduction in response to perturbations produced by wedge prisms is carried primarily by procedural visuomotor recalibration, the correction following dove reversing prisms is largely the result of strategic corrections [9, 31, 32].

To analyse the possible relation of different cognitive processes to the adaptation mechanisms involved in visuomotor recalibration or strategic control during aging, we evaluated young, middle-age, and older participants' performance in a throwing task while donning either wedge or dove prisms, respectively. Then we tested the participants' cognitive performance in 5 cognitive domains including rule acquisition and reversal, recognition memory, visual memory, spatial planning, and spatial working memory from the Cambridge Neuropsychological Test Automated Battery (CANTAB) [33]. We then correlated the participants' performance to their score on the cognitive tests. Based on previous evidence [17, 22], we hypothesized that age-dependent changes in spatial working memory would be correlated with the prism adaptation process only in the strategic dependent task but not as much in the visuomotor recalibration dependent task. The results confirmed our hypothesis by showing a higher correlation between spatial working memory and visuomotor strategic learning than with visuomotor recalibration.

## 2. Methods

### 2.1. Participants and Recruitment

Seventy healthy right-handed volunteers aged 18–85 years (mean 44.26 ± 22.09; 34 females, 36 males) were divided into three groups. The first group consisted of 25 participants aged 18–29 years (mean 22.1 ± 2.4; 13 males, 12 females), the second group consisted of 23 participants aged 30–55 years (mean 41.8 ± 8.0; 11 males, 12 females), and the third group consisted of 22 participants aged 65–85 years (mean 71.5 ± 7.3; 11 males, 11 females). Older participants were recruited from different facilities from a government working program for retired people. The participants reported being healthy at the time of the experiment, had normal or corrected to normal vision, and had no history of mental or neurological injury. The Mini-Mental State Examination (MMSE) was also completed [34], and the participants' scores were within normal values. The MMSE values for each group were as follows: 18–29: 29.32 ± 0.74 SDM; 30–55: 29.17 ± 0.98; 65–85: 27.59 ± 2.17. Participants were informed about the general purpose and procedures of the experiment but were naive about the different experiments. All participants gave informed consent before the experiments in accordance with the Declaration of Helsinki.

### 2.2. Neuropsychological Tests Procedure

Participants performed the following CANTAB tests: the big/little circle (BLC) task to assess comprehension and simple learning; the intra/extradimensional (IED) set shift task to assess rule acquisition, reversal, and attentional set shifting; the delayed matching to sample (DMS) task to assess delayed memory; the Paired Associate Learning (PAL) task to evaluate visual memory; the Stoking of Cambridge (SOC) task to evaluate spatial planning; and spatial working memory (SWM) task to assess spatial working memory. We obtained the following measure outcomes of each neuropsychological test.

### 2.3. BLC

This visual discrimination test is designed to train a subject to follow simple and reversal rules. It is used to familiarize participants with the tests' general procedures and to provide a general idea whether sensorimotor or comprehension difficulties limit collecting valid data from the subject.

### 2.4. IED

It is a test of rule acquisition and reversal. It features (a) visual discrimination and attentional set formation and (b) maintenance, shifting, and flexibility of attention. This test is primarily sensitive to changes to the frontostriatal areas of the brain [35, 36]. We analysed IED EDS errors: errors made in the extradimensional stage of the task are labelled EDS errors, as they have been committed at the stage where the subject is required to make an extradimensional shift. Errors committed at the reversal stage following the EDS stage are not included.

### 2.5. DMS

This perceptual and memory test is sensitive to medial temporal and frontal lobes damage [36]. The responses analyzed were total number of correct responses and total number of trials in which the correct stimulus was selected on the first response.

### 2.6. PAL

This test assesses visual memory and learning and is sensitive to medial temporal lobe impairment. The outcome analyzed was the number of stages completed, which is a key indicator of the subject's overall success.

### 2.7. SOC

This is a test of spatial planning and spatial working memory, which gives a measure of frontal lobe function [35, 36]. This is a computerized version of the tower of London task devised by Shallice [37]. The outcome measure analysed was SOC problems solved in the minimum number of moves, recording the number of occasions upon which the subject has successfully completed a test problem in the minimum possible number of moves. This is a succinct expression of overall planning accuracy in SOC [36].

### 2.8. SWM

This test assesses the participant's ability to retain spatial information and to manipulate remembered items in working memory [38]. The outcome measure analysed was SWM between errors, defined as number of times the subject revisits a box in which a token has previously been found.

### 2.9. Prism Adaptation Procedure

Participants also performed the visuomotor adaptation tasks. The experiment followed a throwing technique previously described [31]. While seated, subjects rested their heads on a chin support that had attached an occluding panel with a 5 × 5 cm window so the subjects could only see with their right eye. This setup occluded the view of the subject's hand during the experiments. Participants viewed the target through the window which had either a clear crystal, a 20-diopter wedge prism that produces a lateral deviation of light to the right, or right-left dove reversing prisms that flip the visual image around the midline. The participants threw clay balls at a target (12 × 12 cm cross drawn on a large sheet of parcel paper) centred at shoulder level 2 meters in front of them. Visual feedback received by participants in each trial allowed them to know the impact location. They were instructed to make only overhand tosses during the whole experiment, to use the right hand, and to throw the balls to the location where they saw the targets. The participants had an unobstructed view of the target during the entire session. During the performance of the task, they were not allowed to look down at their hand as they collected the next ball from a tray located next to them.

### 2.10. General Design of Prism Adaptation Experiments

The two experiments followed three phases as previously described [7, 29, 30]. Displacement prism experiment: subjects threw 26 balls during each condition. During the baseline condition (PRE) subjects did not wear prisms. In the prism condition (PRI) subjects wore 20-diopter wedge displacement prisms. Finally, before starting the last condition (POS), participants had the prisms removed before continuing to throw. Reversal prism experiment: subjects followed the same procedure as previously described, except that during PRI they wore dove reversing prisms. The critical feedback difference between the 20-diopter displacing wedge prism and the reversing prism is as follows: Both prisms led to a 40 cm rightward optical displacement, and to overcome that error in both conditions participants have to make 40 cm leftward corrections. However, while the sign direction of the visual error feedback in the wedge prism is congruent with the real sign direction of the correction, the sign correction of the error feedback in the dove prism is reversed. Therefore, to hit the target during the displacement the participants need to throw where their visual feedback is informing them. By contrast, to hit the target during the reversal of the visual field, the participants would still need to throw to the left, which is incongruent to what their reversed visual feedback is informing them [11]. Prism tasks order was counterbalanced within each group.

### 2.11. Data Analysis

The locations of the impacts were plotted sequentially by trial number (abscissa) versus horizontal displacement (in centimetres) from a vertical line passing through the target centre (ordinate). Impacts to the left of the target were plotted as negative values and impacts to the right were plotted as positive values. Three additional measures were calculated from the collected data. First, based on previous literature where the learning process in visuomotor adaptation contains an “early” phase [17, 22], we searched for the optimal number of consecutive adaptation blocks that resulted in the steepest rate of learning from the first adaptation block (i.e., the slope across the first two, three, four, and *n* trials). Second, an adaptation magnitude was obtained from the PRI condition by subtracting the horizontal displacement to the target on the final throw (26th throw) from that on the initial throw (first throw). Finally, we examined an aftereffect measure which was defined as the impact's horizontal distance to the target on the first throw after removing the prism. To compare both measures between groups we used one-way ANOVA with post hoc tests using the Bonferroni correction. Pearson correlations (*r*) were also computed between participants' performance on each of the neuropsychological tests (please refer to the “measure outcomes of neuropsychological tests” part) and the prism adaptation measure, including (a) “early phase” slope in both perturbations, (b) adaptation magnitude in both perturbations, and (c) aftereffect magnitude of both prism adaptation tasks.

## 3. Results

### 3.1. Cognitive Screening and Age

MMSE one-way ANOVA test showed statistical differences between groups as expected (*F* (2, 67) = 10.440; *p* = 0.000): young group: mean score 29.32, standard deviation (SD) 0.748, and range 28–30; middle-age group: mean score 29.17, SD 0.984, and range 27–30; elderly group: mean score 27.59, SD 2.175, and range 20–30. Tukey post hoc analysis showed significant differences only with the elderly group. It should be noted, however, that these differences were expected, and all groups' scores were higher than the standard cut-off value of 26 out of 30 [34].

### 3.2. Displacement Experiment

**Figure 1:**
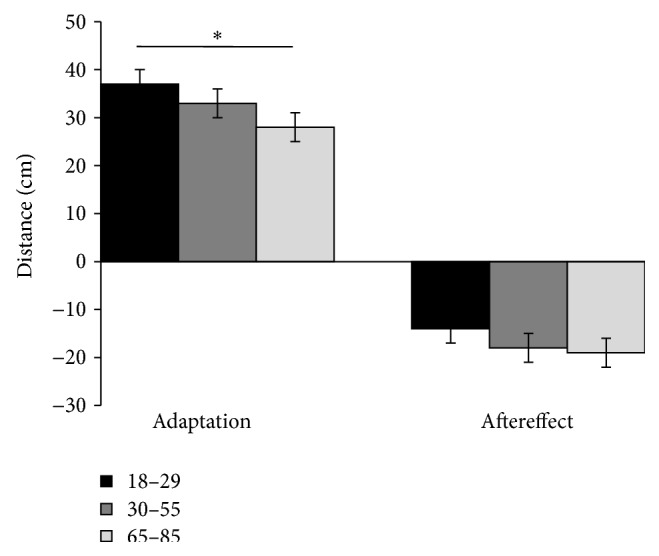
Left: adaptation magnitude in young (black), middle-aged (gray), and older aged (light gray) groups during displacement experiment. Right: aftereffect magnitude in young, middle-age, and elderly group. Bars represent standard errors; asterisk (*∗*) denotes *p* = 0.012.

The adaptation and aftereffect measures in all three groups were different from zero: 18-19: *t* = 18, *p* < 0.01 and *t* = −11.5, *p* < 0.01; 30–55: *t* = 12.1, *p* < 0.01 and *t* = −10.4, *p* < 0.01; 65–85: *t* = 14.4, *p* < 0.01 and *t* = −8.7, *p* < 0.01, respectively. Further analysis showed significant differences between groups in the adaptation magnitude (one-way ANOVA, *F* (2, 67) = 4.754; *p* = 0.012) but only trending differences in the aftereffect (one-way ANOVA, *F* (2, 67) = 2.52; *p* = 0.08) (Figure 1). Bonferroni corrected post hoc tests revealed that the only significant difference was in the adaptation magnitude between the younger and the elderly group (*p* < 0.01).

### 3.3. Reversal Experiment

**Figure 2:**
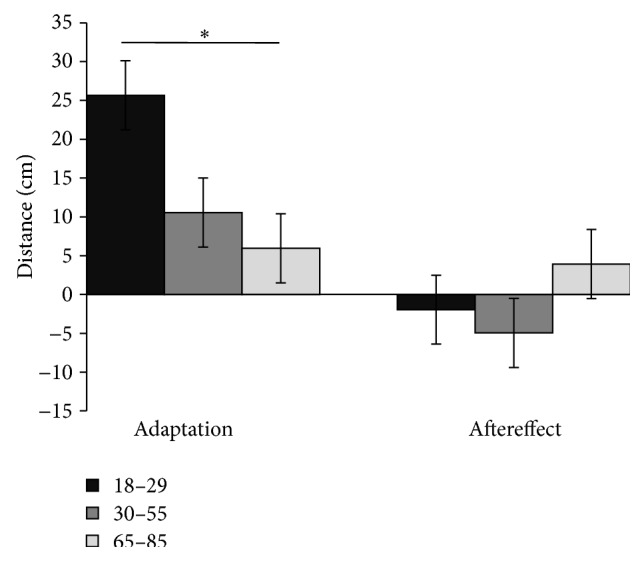
Left: adaptation magnitude in young (black), middle-aged (gray), and older aged (light gray) groups during reversal experiment. Right: aftereffect magnitude in young, middle-age, and elderly group. Bars represent standard errors; asterisk (*∗*) denotes *p* = 0.024.

There were also significant differences between groups in the adaptation magnitude in the reversal experiment (one-way ANOVA, *F* (2, 67) = 3.331; *p* = 0.024) but not in the aftereffect (Figure 2, left and right, resp.). Post hoc test using the Bonferroni correction revealed that young and elderly groups were the only statistically significantly different ones in the reversal prism adaptation magnitude (*p* < 0.05).

### 3.4. Visuomotor Correlations with Age

The analysis showed significant negative correlations between age and the magnitude of the adaptation in both displacement (*r* = −0.37, *p* = 0.002) and reversal prism conditions (*r* = −0.33, *p* = 0.006). We also found a weak correlation between age and aftereffect in the displacement condition (*r* = 0.24; *p* = 0.05), but not in the reversal condition.

### 3.5. Learning Slopes

Table 1 shows the slopes for the first ten adaptation trials. We found that the steepest rate of learning that corresponded to the early adaptation period occurred during the first two throws in the displacement prism task; however, this was not the case for the reversal task.

### 3.6. Behavioural Correlations Analysis

The adaptation magnitudes from both prism tasks were also correlated with the CANTAB cognitive tasks.  Table 2 shows the results of the correlation analysis between the neuropsychological tests and the adaptation magnitudes. Significant negative correlations were found between errors in SWM and the adaptation magnitudes of the reversing prism (*r* = −0.49, *p* = 0.001) and the displacement prism (*r* = −0.38, *p* = 0.01). None of the other correlations were significant.

Since age also had a significant correlation with SWM between errors (*r* = 0.621, *p* < 0.001), we ran a partial correlation to control the effect of age in the relationship between visuomotor learning and cognitive processing. The result shows that when we control age on the relationship between reversal adaptation and SWM between errors, there is still a significant partial correlation (Table 3) (*r* = −0.39, *p* = 0.001). However, the same analysis showed that the partial correlation was not significant between SWM and displacement adaptation while controlling for age (*r* = −0.27, *p* = 0.08).

## 4. Discussion

The aim of this study was to test the hypothesis that age-dependent cognitive deterioration correlated with strategic visuomotor learning capacity. Our results confirmed this hypothesis by showing a significant correlation between spatial working memory scores and strategic visuomotor learning. This correlation survived even after correcting for aging, contrary to the correlation with visuomotor recalibration that did not survive the correction. Following is a detailed discussion of our results.

### 4.1. Visuomotor Adaptation and Aging

Previous studies have shown that aging results in a decrease in motor learning under congruent feedback conditions [5, 8, 16, 18]. Our results expanded these observations to visuomotor learning carried under reversed feedback conditions, confirming recent findings that suggested a variable adapting rate and null aftereffects in this type of adaptation [11, 31, 32].

### 4.2. Correlations between Cognitive Functions and Visuomotor Adaptation

Visuomotor adaptation involves the recalibration of a sensorimotor mapping. Although this kind of adaptation involves implicit sensorimotor processes [6], there is evidence that modification of these representations engages cognitive resources [9–13, 17, 22]. For example, previous studies using computerized reaching tasks have found significant correlations between learning rate and spatial working memory processes [3, 17] and more automatic late learning stage [15, 17, 21]. Here we did not find correlations between the initial learning rate and working memory, probably due to the nature of our motor task and the number of trials that participants were required to perform in our experiments (26 in our study compared to several adaptive blocks in other studies [17, 22]). However, we used the adaptation magnitude measure of both perturbations to examine correlations with individual performance during the CANTAB cognitive tasks. Using these measures we found that both perturbations correlated with the number of errors in SWM. These findings are consistent with previous reports that suggest (a) a specific contribution of spatial working memory processes during a rotation task and (b) that a failure to effectively engage spatial working memory processes contributes to age-related deficits in visuomotor rotation adaptation [17, 22].

Given that the SWM errors had a positive correlation with age it was important to account for the age variance in the analysis with the adaptation measure. In view of this, we carried out a partial correlation analysis controlling for age. Using this analysis we found that the correlation between displacement adaptation and SWM did not survive, but the correlation with the reversal adaptation still remained. The relevance of this study, compared to previous works that have reported the importance of SWM processes during visuomotor learning, is the nature of our motor task and perturbations. Anguera et al. [17] formulated the question if the relationship between visuomotor adaptation and SWM is task specific or whether SWM processes play a role in other motor learning tasks. Visuospatial working memory capacity has been reported to predict the organization of explicitly acquired motor sequences [39], supporting the possibility that it may generalize to other types of motor tasks. Our study provides additional support to the hypothesis that SWM plays a role in a specific motor task, specifically the visuomotor learning during prism adaptation.

We propose that SWM processes are preferentially involved during the adaptation to the reversed visual field. A successful realignment process with reversing prisms would require the brain to reverse the feedback rule used to update motor commands [40]. Previous works from our group and others [11, 13, 14, 41] suggest that subjects show an impairment to adapt to a reversed visual field, due to significant deleterious effects on visuomotor strategic learning. Our results support this last affirmation and allow us to suggest that failure to effectively engage spatial working memory processes could result in a decrease of visuomotor adaptation. This deficit would start in middle-age group and would keep progressing across aging, a finding that is consistent with reports showing how age-related cognitive changes can be detected during middle age [42].

### 4.3. How Is SWM Involved during Prism Adaptation?

Current views of learning have posited a role of working memory in both error temporary storage and error processing [42]. For motor learning this would suggest that the storage of movement errors and the computation of an internal model also take place in working memory processes [43]. This view supports previous proposals that working memory is related to the learning rate in a motor learning task in older adults [8, 44]. Our data confirms the participation of SWM process during visuomotor adaptation, specifically during a throwing task using a reversal prism adaptation paradigm. SWM could be used to maintain the spatial localization of the throws' impacts in relation to the target, as well as in the further evaluation of how those impacts evolve across trials. Since the evaluation of these processes requires the implementation of a strategic approach during the reversing prism, then its influence could be larger than during the procedural learning, which does not require further cognitive processing [10–13]. This interpretation agrees with Willingham [45] who suggested that cognitive resources are recruited from trials allowing the error information to be integrated to update visuomotor maps for the subsequent trial. It should be noted, however, that this process is probably different from those involved in error detection per se, since those processes have been suggested to be independent of working memory; for example, the parametric manipulation of working memory yields similar imaging results in the prefrontal cortex in both error and error-free trials, suggesting a minimum contribution of the error detection system in those areas showing working memory parametric activity variations [46].

## 5. Conclusion

Here we show that variations in spatial working memory integrity correlate with visuomotor learning capacity. While this correlation shows a significant decrease when age is accounted for in visuomotor realignment, it is still significant for visuomotor learning depending on strategic control. These results support the hypothesis that, contrary to the other cognitive processes evaluated, spatial working memory is a fundamental component of the cognitive processes subserving visuomotor learning that depends on strategic control.

## Figures and Tables

**Table 1:** Adaptation slopes during displacement and reversal adaptation phase.

Throw	1st 2	1st 3	1st 4	1st 5	1st 6	1st 7	1st 8	1st 9	1st 10
Displacement	−5.33	−4.94	−3.98	−3.68	−3.37	−2.99	−2.73	−2.61	−2.49
Reversal	−1.30	−1.82	−3.02	−2.08	−2.05	−2.00	−1.86	−1.62	−1.46

**Table 2:** Pearson's correlation *r* values between each CANTAB variable and adaptation magnitude for the displacement and reversal tasks. Asterisk (*∗*) denotes significant correlations at the 0.001 level.

Variable	Displacement	Reversal
	*r*	
IED EDS errors	−.26	−.12
DMS-total correct	.11	.20
PAL complete stages	.22	.15
SOC solved minimum	.27	.28
SWM between errors	**−**.38^**∗**^	**−**.49^**∗**^

**Table 3:** Partial correlation between SWM between errors and displacement and reversal adaptation, respectively, while controlling the effect of age. Asterisk (*∗*) denotes that correlation is significant at the 0.05 level.

Control variable	Variable	Displacement	Reversal
None	SWM between errors	**−**.33^**∗**^	**−**.47^**∗**^
Age (years)	SWM between errors	−.27	**−**.39^**∗**^
